# What Patient Factors Predict Physicians’ Decision Not to Treat Latent Tuberculosis Infection in Tuberculosis Contacts?

**DOI:** 10.1371/journal.pone.0076552

**Published:** 2013-09-30

**Authors:** Claudia C. Dobler, Queenie Luu, Guy B. Marks

**Affiliations:** 1 Woolcock Institute of Medical Research and Central Clinical School, University of Sydney, Sydney, Australia; 2 Department of Respiratory Medicine, Liverpool Hospital, University of New South Wales, Sydney, Australia; Wadsworth Center, United States of America

## Abstract

**Objective:**

The study aimed to determine factors that are associated with physicians’ decision to offer treatment for latent tuberculosis infection (LTBI) in contacts of patients with tuberculosis.

**Methods:**

We performed a nested case-control study in a cohort of contacts of patients with pulmonary tuberculosis who had a tuberculin skin test (TST) ≥ 10 mm. Cases were those who were offered treatment for LTBI. Controls were randomly selected from those who were not offered treatment for LTBI by the reviewing physician. Odds ratios were estimated by multivariate logistic regression.

**Results:**

There were 195 cases and 279 controls. The following factors were significantly (positively or negatively) associated with being offered LTBI treatment in the multivariate analysis: female gender (OR 2.9; 95% CI 1.6–5.5), TST conversion (OR 3.9; 2.0–7.9), TST > 20 mm (OR 4.1; 1.8–9.1, for TST of 21–30 mm and OR 7.9; 2.6–23.8, for TST >30 mm), sputum smear positive index case (OR 12.7; 4.5–36.1), being overseas-born and immigration more than 2 years ago (OR 0.1; 0.06–0.3), being a health care worker (OR 0.2; 0.1–0.6), being a non-household contact of the TB index case (OR 0.3; 0.2–0.6) and age >35 years (OR 0.2; 0.1–0.5 for age 35 to 54.9 years and OR 0.04; 0.01–0.2 for age ≥55 years). Previous BCG vaccine and chest x-ray findings were not significantly associated with physicians’ decision to offer treatment for LTBI.

**Conclusions:**

Most factors that influenced physicians’ decisions on treatment for LTBI were based on evidence of an association with risk of developing TB or risk of having an adverse reaction to treatment for LTBI. However, the decreased likelihood of offering treatment for LTBI to people born overseas, men and health care workers, was apparently not based on any evidence of risk. Efforts should be made to ensure that these groups are given access to treatment for LTBI.

## Introduction

Treatment of latent tuberculosis infection (LTBI) is an important component of tuberculosis (TB) control strategies, especially in settings where TB is not endemic [1]. In developed countries screening for and treatment of LTBI is implemented among high risk groups such as contacts of TB cases, HIV+ persons and those in whom TNF-alpha blocker therapy is to be commenced [1]. Yet, in a recently conducted cohort study examining the risk of active TB and the prevalence of LTBI among 14,371 contacts of patients with TB in Sydney, Australia, we found that only 9.5% of TB contacts with evidence of LTBI (409/4,351) received treatment for LTBI [2], [3]. We were interested to examine factors associated with physicians’ decision to treat or not treat LTBI among these contacts.

The decision to give preventive therapy for LTBI is a complex one in which risk of toxicity from treatment, risk of progression to active TB, and risk of fatal outcome of either toxicity or active TB must be considered. The estimated risk of developing TB depends on the probability that true LTBI is present and the risk of progression from LTBI to active TB if an individual is infected. The probability that LTBI is present in an individual is a function of the pre-test probability of LTBI and the result of the test for LTBI, that is the tuberculin skin test (TST) or an Interferon Gamma Release Assay (IGRA).

The risk of transmission of TB between a patient with the disease (the index case) and a contact of that patient, and thus the pre-test probability of LTBI in the contact, depends on the infectiousness of the index case, the proximity of the contact to the index case and the duration of exposure. The most infectious TB patients are those with positive sputum smears [4] and the contacts most at risk are usually household contacts because of their proximity to the index case and their duration of exposure in a closed environment [5]. The risk of progressing to active disease after infection decreases with time after infection [6] and is increased in the presence of certain medical conditions including HIV infection, silicosis, diabetes, renal replacement therapy, gastrectomy, smoking, underweight and TNF-alpha-blocker treatment [7]–[10].

Further, the risk of side effects associated with treatment of LTBI is relevant to the decision to offer treatment for LTBI. The most common serious adverse effect is drug-induced hepatotoxicity, the risk of which increases with age [11]–[13].

The Sydney cohort of 14,371 contacts of TB patients, of whom only a small proportion was given treatment for LTBI, comprised contacts at different levels of risk for developing TB and included household as well as non-household contacts. Using a nested case-control study design, we aimed to determine patient characteristics associated with physicians’ decision to offer, or not to offer, treatment for LTBI among TB contacts with a TST ≥10 mm.

## Methods

### Study setting and population

The study was conducted at the TB clinic of a metropolitan tertiary hospital in Sydney, Australia, which manages approximately 10% of all TB patients that are notified in Australia.

The process for deciding on a recommendation for treatment of LTBI in this TB Clinic entailed two stages. In the first stage the attending TB physician reviewed the TST result and the chest x-ray, together with demographic and clinical information that was recorded in the patient’s file. Based on this review some members of the cohort were invited to attend a clinic appointment with the attending physician. It was at this clinic appointment that a final decision to offer, or not to offer, treatment of LTBI was made.

### Study design

A nested case-control study was conducted among contacts of patients with pulmonary TB who were screened at this clinic between 01/01/2000 and 31/12/2010 and who had a TST ≥ 10 mm. All cases and controls were part of a cohort of 14,371 contacts of patients with TB in Sydney, Australia [2]. Cases were members of the study cohort who were offered treatment for LTBI- independently of whether they accepted treatment or not. Controls were members of the study cohort who were not offered treatment for LTBI; they were randomly selected (using a random number generator) from a computer-based database that contained information on all patients who had been reviewed (in person or via case notes and chest x-ray) at the TB clinic during the study period.

Study data were obtained from a clinical database containing details for all patients seen at the TB clinic and from their medical records.

### Study factors

Two researchers extracted data from both cases and controls with 20% of all cases and controls being reviewed by both researchers to ensure a uniform approach to data extraction. Discrepancies were resolved by consensus. We extracted data on age, sex, TB incidence in country of birth, TST size, TST conversion, history of previous TST, contact type, time since immigration to Australia for overseas born people, chest x-ray findings, BCG vaccination status, health care worker status, disease site of the index case and sputum smear result of the index case.

### Definitions

“TST conversion” was defined as an initial TST ≤10 mm with an increase in TST size of ≥6 mm and a TST ≥10 mm at follow up [14]. “TST in mm” was defined as the measured diameter of the skin induration (using a millimeter ruler) 48 to 72 hours after the skin test had been administered. “Health care worker” referred to doctors, nurses, allied health professionals, students on clinical practice, laboratory staff and support staff who may have had contact with (TB) patients as a result of their workplace activities. An “abnormal chest x-ray” was defined as a chest x-ray with any lung or pleural abnormality reported by the reviewing physician.

### Sample size calculation

The study had 80% power to detect an odds ratio (OR) of 1.7 for an association between offering LTBI treatment and a specific patient characteristic based on 195 cases and 279 controls and an assumed prevalence of the risk factors of 0.30 in the controls. Hence, the study was adequately powered to detect clinically relevant odds for factors influencing physicians’ decision to offer LTBI treatment. The number of controls was capped at 279, because further increase in the number of controls was only associated with a minimal improvement in the detectable OR.

### Data analysis

Associations between demographic and clinical characteristics and case versus control status were estimated as Odds Ratios (ORs) with 95% confidence intervals (95% CIs) using univariate and multivariate logistic regression. The variables for inclusion in the multivariate model were chosen based on plausibility rather than their statistical association with the dependent variable in the univariate analysis. As the outcome measured was a physician’s decision to offer treatment for LTBI, based on information that was available at the time of clinical review, missing information would have been taken into account by the physician and therefore potentially contributed to the outcome (decision). Variables with missing values where therefore included in the analysis. Statistical analyses were carried out using SPSS Statistics 20 (IBM, Armonk, NY, USA)

### Ethical considerations

The study protocol was approved by the the NSW Population & Health Services Research Ethics Committee. The requirement for written or verbal patients’ consent for this study was waived by the above ethics committees because existing (clinical) data sources were used.

## Results

### Description of the study population

During the study period 195 contacts of patients with pulmonary TB were offered treatment for LTBI. Of the 195 cases who were offered treatment for LTBI, 14 (7.2%) declined treatment. Those 14 patients were included in the analysis as cases, as the outcome of interest was the physician’s decision to offer treatment rather than the patient’s decision to accept or decline. From a pool of 2,147 contacts who were not offered LTBI treatment by the reviewing physician 279 controls were randomly selected. The randomly selected controls were representative of the overall patient group (no significant differences were found for any of the variables included in the multivariate analysis). Of the 279 controls, 50 had been reviewed in person by a physician at the TB clinic. In the remainder the decision not to offer LTBI treatment was based on a physician’s review of the medical file.

Six physicians were involved in reviewing patients and case files with chest x-rays. All physicians were pulmonary specialists who were seeing adults as well as children.

Characteristics of cases and controls are shown in Table 1. The median age (interquartile range) of cases and controls was 17.9 years (17.4) and 38.0 years (20.0) respectively. Fifty-nine per cent of the cases were female, and 52% of the controls were female (p = 0.106). The proportion of overseas-born patients was 44% (85 out of 194 with information on country of birth) among cases and 83% (227 out of 274) among controls (p<0.001).

**Table 1:** Patient characteristics associated with physicians’ decision to offer LTBI treatment.

Factor	Cases	%	Controls	%	Treatment offered			
					unadjusted Odds Ratio (95% CI)	P value	adjusted Odds Ratio (95%CI)	P value
**Sex**						**0.1**		0.001*
Male	79	41	134	48	1.00 (Ref)		1.0	
Female	116	59	145	52	1.4 (0.9 to 2.0)		2.9 (1.6 to 5.5)	
**Age, years**						<0.001*		<0.001*
0 to 6.9	25	13	1	0	35.9 (4.7 to 271.0)	0.001*	12.5 (1.2 to 135.1)	0.04*
7 to 17.9	73	37	9	3	11.6 (5.5 to 24.8)	<0.001*	4.7 (1.8 to 12.2)	0.001*
18 to 34.9	69	35	99	35	1.0 (Ref)		1.0	
35 to 54.9	25	13	127	46	0.3 (0.2 to 0.5)	<0.001*	0.2 (0.1 to 0.5)	<0.001*
≥ 55	3	2	43	15	0.1 (0.03 to 0.3)	<0.001*	0.04 (0.01 to 0.2)	<0.001*
**Country of birth**						<0.001*		<0.001*
Australian born	109	56	47	17	1.0 (Ref)		1.0	
Overseas born, immigrated < 2 years ago	8	4	2	1	1.7 (0.4 to 8.4)	0.5	0.6 (0.1 to 4.6)	0.6
Overseas born, immigrated > 2 years ago	77	39	225	81	0.1 (0.1 to 0.2)	<0.001*	0.1 (0.06 to 0.3)	<0.001*
No info	1	1	5	2	0.1 (0.01 to 0.8)	0.02*	0.1 (0.01 to 2.1)	0.2
**Health care worker**						<0.001*		0.002*
No	179	92	203	73	1.0 (Ref)		1.0	
Yes	16	8	76	27	0.2 (0.1 to 0.4)		0.2 (0.1 to 0.6)	
**TST in mm**						0.04*		<0.001*
10 to 15	70	36	119	43	1.0 (Ref)		1.0	
16 to 20	50	26	87	31	1.0 (0.6 to 1.5)	0.9	1.5 (0.7 to 3.3)	0.3
21 to 30	57	29	54	19	1.8 (1.1 to 2.9)	0.02*	4.1 (1.8 to 9.1)	0.001*
>30	18	9	19	7	1.6 (0.8 to 3.3)	0.2	7.9 (2.6 to 23.8)	<0.001*
**TST conversion^#^**						0.002*		<0.001*
no	132	68	224	80	1.0 (Ref)		1.0	
yes	63	32	55	20	0.1 (0.4 to 0.5)		3.9 (2.0 to 7.9)	
**BCG**						<0.001*		**0.2**
no	99	51	51	18	1.0 (Ref)		1.00.	
yes	84	43	217	78	0.2 (0.1 to 0.3)	<0.001*	0.5 (0.2 to 1.1)	0.09
no info	12	6	11	4	0.6 (0.2 to 1.4)	0.2	0.9 (0.2 to 4.2)	0.9
**Chest x-ray**						**0.2**		**0.5**
normal	179	92	240	86	1.0 (Ref)		1.0	
abnormal	13	7	33	12	0. (0.3 to 1.0)	0.06	0.6 (0.2 to 1.5)	0.2
No info	3	2	6	2	0.7 (0.2 to 2.7)	0.6	1.0 (0.1 to 15.2)	0.999
**Contact type**						<0.001*		0.004*
Household contact	115	59	88	32	1.0 (Ref)		1.0	
Non-household contact	80	41	159	57	0.4 (0.3 to 0.6)	<0.001*	0.3 (0.2 to 0.6)	0.004*
No info	0		32	11	not estimable		not estimable	
**AFB sputum smear of index case**						0.001*		<0.001*
negative	13	7	52	19	1.0 (Ref)		1.0	
positive	176	90	217	78	3.2 (1.7 to 6.1)	<0.001*	12.7 (4.5 to 36.1)	<0.001*
No info	6	3	10	4	2.4 (0.7 to 7.8)	0.1	3.5 (0.4 to 28.6)	0.2

# TST conversion was defined as an initial TST ≤10 mm with an increase in TST size of ≥6 mm and a TST ≥10 mm at follow up.

### Factors associated with being offered treatment for LTBI

Univariate analysis showed that age<18 years, TST 21–30 mm, TST conversion, a history of BCG vaccination and being a contact of an index case who was sputum smear positive were significantly positively associated (p<0.05) with being offered treatment for LTBI (Table 1). Age >35 years, being overseas-born and having immigrated to Australia >2 years ago, being a health care worker and being a non-household contact were significantly negatively associated (p<0.05) with being offered treatment for LTBI. In the univariate analysis sex, having a TST >20 mm, and having abnormal chest x-ray findings were not significantly associated with being offered treatment for LTBI.

Multivariate analysis was performed with adjustment for sex, age, country of birth, health care worker status, TST in mm, TST conversion, BCG status, chest x-ray findings, contact type and the result of the AFB sputum smear in the index case. The outcome for the following variables differed in the multivariate analysis compared to the univariate analysis: females were more likely to receive treatment, previous BCG vaccination was no longer significantly associated with being offered (or not offered) treatment for LTBI and a TST >30 mm was found to be significantly associated with the likelihood of receiving treatment for LTBI. Table 1 shows the unadjusted and adjusted odds ratios with 95% confidence intervals for the association between personal and clinical characteristics and the decision to offer treatment for LTBI.

## Discussion

This nested case-control study that was conducted in a cohort of TB contacts with TST ≥ 10 mm in Sydney, Australia, identified a number of factors that were significantly associated with an increased or decreased likelihood of being offered treatment for LTBI by the reviewing physician. Most factors that influenced physicians’ decisions on treatment for LTBI were based on well-known evidence of an association with risk of developing TB or risk of having an adverse reaction to treatment for LTBI. However, the reasons for some associations, such as the decreased likelihood of offering treatment for LTBI to people born overseas, men and health care workers, were less evident.

The policy directive on treatment for LTBI during the study period in New South Wales, the Australian state where the study was performed, stated that LTBI treatment should be considered in the case of: recent TST converters (within two years), close contacts of a sputum smear positive source case who have a strongly positive TST and are <35 years old irrespective of previous BCG status, close contacts < five years of age of a sputum smear positive case irrespective of TST status and children <16 years old with a strongly positive TST even if not a contact of a known smear positive case [15].

As expected, our study confirmed a correlation between physicians offering treatment for LTBI and factors that are known to be associated with an increased risk of developing TB, such as being a household contact, having a TST conversion, having a strongly positive TST, being the contact of an index case who is sputum smear positive and young age [5], [16], [17].

Our data showed a significant association between a history of BCG vaccination and reluctance to offer treatment for LTBI in univariate analysis. However, this association was not confirmed in the multivariate analysis. The discrepancy between the univariate and multivariate model finding can be explained by a significant association between a history of BCG and being overseas born. Of overseas born people, 252 out of 302 (83%) had a BCG versus 44 out of 143 (31%) of Australian born people (p<0.001). As overseas born people were less likely to be offered LTBI treatment, this explains why people with BCG were significantly less likely to be offered treatment in the univariate analysis.

A US study found that a significant proportion of international medical graduates who had been BCG vaccinated themselves and had a positive TST, attributed the positive TST to the BCG vaccination and believed that BCG would protect them against TB [18]. BCG vaccination has been shown to reduce the risk of developing TB [5], [19]. However, protection from BCG vaccination is likely to wane with time since vaccination, and there is no clear evidence that BCG provides protection beyond ten years after vaccination [20]. Further, BCG vaccinations received in infancy have no significant influence on tuberculin sensitivity (and thus size of the TST) later in life [21], [22]. Hence, the effect of TST reaction size on the risk of developing TB has been shown not to be affected by the presence of a BCG scar [23]. Studies have found that positive TSTs in BCG-vaccinated children from TB-endemic countries likely represent true LTBI [24], [25]. Guidelines by the American Thoracic Society (ATS)/Centers for Disease Control and Prevention recommend that clinicians should ignore history of BCG vaccination when interpreting the TST in adults [1]. The study finding regarding BCG vaccination in the multivariate analysis is thus consistent with current evidence and recommendations.

The observed decreased likelihood of offering treatment for LTBI in contacts aged >35 years can be explained by the increased risk of drug-induced hepatitis in this age group [11], [13].

The finding that people born overseas, health care workers and men (in the multivariate analysis) were less likely to be offered treatment for LTBI was unexpected. One of the challenges in making a decision about treatment for LTBI in contacts born in countries where TB is endemic is uncertainty about whether infection is likely to be due to the recent contact with a TB patient or due to remote past infection. This is relevant because the risk of progressing to active TB is greatest in the first two years after infection and significantly decreases over time [6], making treatment for LTBI less beneficial if the infection was acquired many years ago. However, in the Sydney TB contact cohort study we found that overseas-born contacts had a higher risk of developing active TB than Australian-born contacts (after adjustment for age, gender, TST size and whether preventive treatment was given) with odds ratios of 3.35 (95% CI 1.84–6.10), 3.64 (95% CI 2.48–5.35) and 5.79 (95% CI 4.08–8.21) in overseas-born contacts from countries with a TB incidence of <10, 10–99 and ≥100 per 100 000 population respectively [3]. Hence, the observed lower likelihood of offering treatment for LTBI to overseas-born contacts, compared to Australian-born contacts, is not justified by the available evidence.

Univariate analysis did not find a sex difference for the likelihood of being offered treatment for LTBI, whereas multivariate analysis found that males were less likely than females to be offered treatment. There were no significant associations between sex and BCG status (p = 0.66) or sex and TST>20 mm (p = 0.693). The reason for the discrepancy between the univariate and multivariate model finding was that being a woman was significantly associated with being a healthcare worker (p<0.001). As healthcare workers were less likely to be offered LTBI treatment this masked the sex difference in the univariate analysis.

A number of studies have reported an increased risk of hepatotoxicity in women on treatment for active TB [26]. However, there is currently no clear evidence indicating an overall increased risk of hepatotoxicity in women on treatment for LTBI [26]. Pregnant women in the third trimester and in the first three months of the postpartum period may have an increased risk to develop drug-induced hepatitis on LTBI treatment [27]. The fact that men were less likely to be offered LTBI treatment (in multivariate analysis) can thus not be explained with an increased risk of adverse events in men.

Rates of notification of TB are higher in men, especially in low income countries where twice as many men are notified with TB as women [28]. In Australia the male to female ratio for TB notifications has been 1:0.8 in recent years [29]. Data indicating higher rates of TB among men, have been the subject of much debate, particularly when obtained from developing countries, because socioeconomic and cultural factors could lead to barriers in accessing health care and may cause under-notification in women [30]. However, a Mexican study that used passive case finding and included over 8000 persons with a cough for 15 days or more- of which 56% were women- confirmed higher rates of bacteriologically proven pulmonary TB among men than women [31]. This was found to be the result of both higher rates of reactivation of LTBI as well as higher rates of recent TB transmission among men (based on molecular epidemiological techniques). While there is no clear evidence for an increased risk of TB reactivation in women, it is possible that physicians’ preconceptions that men would be less likely to accept treatment for LTBI and more likely to not adhere to the prescribed course of LTBI treatment, led to a gender bias in LTBI treatment offer in our study. However, previous studies (including from the current study setting) on acceptance and completion of treatment for LTBI do not in general support these assumptions [32]–[34].

Health care workers have been found to be less likely to accept treatment for LTBI compared to non-health care workers in previous studies [34], [35]. Physicians seem to be sceptical about taking treatment for LTBI. A US study showing that only 25% of physicians with evidence of LTBI had actually received treatment for LTBI [36]. It is possible that physicians’ reluctance to accept treatment of LTBI themselves is the reason for the decreased likelihood that they will offer this treatment to other health care workers. However, health care workers could potentially transmit the infection to their patients (including children and immunosuppressed patients) if they develop active TB. Hence, efforts should be made to increase the rate of treatment for LTBI among health care workers.

The reason why 33 people with a positive TST and an abnormal chest x-ray did not receive LTBI treatment is unclear. As the multivariate analysis failed to show an association between an abnormal chest x-ray and being offered LTBI treatment this cannot be explained by any of the measured confounders. Only one of these patients had a history of previous TB and had received treatment for TB. Considering that patients with chest x-ray abnormalities are at an increased risk of TB reactivation [37], this finding is counterintuitive.

This study has several limitations. Physicians’ decisions about treatment of LTBI at this major TB clinic in Sydney were not necessarily representative of other TB clinics in Australia. However, we propose that the factors that were associated with physicians’ reluctance to offer treatment for LTBI in this setting will help to elucidate barriers to the provision of treatment for LTBI in other settings where rates of treatment for LTBI are low. As the study was based on retrospective chart review, it is possible that, at the time of making a decision about treatment for LTBI, the reviewing physician had additional information available, which was not written down in the chart. While bias due to data extraction- as in any retrospective chart review study- cannot be excluded, we attempted to minimise this risk by using two independent reviewers for both cases and controls. As over 80% of controls were not seen in person by the reviewing physician (in contrast to cases who were all reviewed at the TB clinic), and written statements on whether treatment for LTBI was offered or not were consistently included in the clinical files, the risk for misclassification would have been relatively small. The reviewing physician was not included as an explanatory variable, because patients in whom the decision for or against LTBI treatment was considered to be controversial by the reviewing physician were discussed at a meeting with all physicians conducting TB clinics, and consensus was sought.

## Conclusions

Most factors that influenced physicians’ decisions on treatment for LTBI were based on evidence of an association with risk of developing TB or risk of having an adverse reaction to treatment for LTBI. However, the decreased likelihood of offering treatment for LTBI to people born overseas, men and health care workers, apparently was not based on any evidence of risk. Efforts should be made to ensure that these groups are given access to treatment for LTBI.
